# Chest CT Diagnosis of COVID-19: Accuracy using CO-RADS and CT-Involvement Scoring

**DOI:** 10.5334/jbsr.2342

**Published:** 2021-04-05

**Authors:** Brecht Van Berkel, Jan Vandevenne, Kristof Coursier, Vincent Alberts, Jan Van Offenwert, Jan Verduyckt, Martijn Grieten, Wim Siemons, Geert Verswijvel

**Affiliations:** 1 Department of Radiology, Ziekenhuis Oost-Limburg, Schiepse Bos 6, 3600 Genk, Belgium; 2 Department of Radiology, University Hospitals Leuven, Herestraat 39, 3000 Leuven, Belgium; 3 Faculty of Medicine, University of Hasselt, Hasselt, Belgium; 4 Department of Radiology, University Hospital Brussels, Laarbeeklaan 101, 1090 Jette, Belgium; 5 Department of Radiology, Antwerp University Hospital, Wilrijkstraat 10, 2650 Edegem, Belgium

**Keywords:** COVID-19, CT, RT-PCR, CO-RADS, Chest, Infection

## Abstract

**Objectives:**

Both Reporting and Data System (CO-RADS) and CT-involvement scores (CTIS) have been proposed for evaluation of COVID-19 on chest CT. The purpose of this single-center, retrospective study was to evaluate both scoring systems to diagnose COVID-19 infection in a high-prevalence area.

**Materials and Methods:**

Chest CT datasets (n = 200) and available reverse transcription polymerase chain reaction (RT-PCR) on nasopharyngeal swab were included. CT scans were assigned to four ‘imaging groups’ after scoring for both CO-RADS and CTIS. Diagnostic accuracy of chest CT was calculated respectively using RT-PCR and clinical diagnosis as gold standards: False-negatives and false-positives of chest CT regarding RT-PCR were studied in more depth using the medical files.

**Results:**

The ‘imaging group’ including CO-RADS 4/5 scores reached the highest diagnostic values for COVID-19 considering either the initial RT-PCR or the final clinical diagnosis as the standard of reference: accuracies of 172/200 (86%) to 181/200 (90.5%), sensitivities of 60/80 (88.2%) to 70/79 (88.6%), specificities of 112/132 (84.9%) to 111/121 (91.7%), negative predictive values (NPV) of 112/120 (93.3%) to 111/120 (92.5%), respectively. False-negative CTs regarding RT-PCR were mainly explained by imaging very early in the disease course (5 out of 8 cases) or COVID-19 infection with no/minor respiratory symptoms (3 out of 8 cases).

**Conclusion:**

Assessing chest CT using CO-RADS is a valuable diagnostic approach for COVID-19 infection in a high-prevalence area, with a higher accuracy than CTIS.

## Introduction

Coronavirus disease 19 (COVID-19) swiftly spread from Wuhan, China, to other Asian countries, Europe, Northern America, and globally [1,2]. A reverse transcription polymerase chain reaction (RT-PCR) lab test on nasopharyngeal swab was quickly developed and gained widespread use [3,4]. This test was reported to have very high specificity but relatively lower sensitivity, with clinical case review resulting in 11–25% false negative results [5,6,7,8]. Rapidly, the radiological community recognized the potential of chest CT to diagnose COVID-19, as COVID-19 infection with pulmonary involvement resulted in typical changes of lung parenchyma such as ground glass opacities with a peripherally distribution in multiple lobes. [9,10,11]. The Dutch Radiological Society developed the COVID-19 Reporting and Data System (CO-RADS), which is a categorical assessment scheme on chest CT, from 1 (very low) to 5 (very high) of the likelihood for COVID-19 infection in patients with moderate to severe symptoms [12]. The extent of pulmonary involvement on chest CT using CT involvement scores (CTIS) also has been correlated with the severity of COVID-19 [13,14]. The difference between CO-RADS and CTIS is that the first looks for typical patterns of COVID-19 infection rather than the extent of these patterns, whereas the second looks for the extent of these typical signs. As high CTIS scores are known to be associated with severe COVID-19 disease, we thought it would be interesting to investigate whether CTIS can also be used as a diagnostic tool in patients with mild to severe COVID-19. The primary goal of this single-center, retrospective study is to evaluate the accuracy of chest CT to detect COVID-19 using respectively CO-RADS and CTIS and to see if these scores can be equally used for the diagnosis of COVID-19 infection.

## Materials and Methods

### Inclusion criteria

Approval for this study was obtained by the internal review board. Chest CT performed in a clinical setting at our hospital from March 7–April 13, 2020, were included. Studies without an associated RT-PCR test for COVID-19 within a 48-hour time interval before or after CT were excluded. CT-scans included inpatient and outpatient studies. The scanners and scanning parameters were: Somaton Force (110 KvP, 76 mAs, 300 FOV, collimation 192 × 0.6 mm) Somaton Emotion (110 KvP, 70 mAs, 300 FOV, collimation 16 × 0.6 mm), and Somaton Definition AS (120 KvP, 78 mAs, 300 FOV, collimation 64 × 0.6 mm) (Siemens healtineers, Erlangen, Germany). The scanning protocols were one of three standard protocols according to the clinical request, that is, without intravenous contrast and contrast-enhanced either in the pulmonary arterial phase (only on Somaton Force scanner) or in the systemic arterial phase. Images were reconstructed with a slice-thickness of 1 mm in mediastinal and lung kernels. All images were reviewed in a standard clinical Picture Archiving and Diagnostic System workstation (IMPAX Agility, Agfa Healthcare, Mortsel, Belgium). The results of RT-PCR performed in all patients with respiratory symptoms or other symptoms suggestive of COVID-19 were retrieved from the patient medical files. Likewise, the final clinical diagnosis whether or not a patient was considered COVID-19 positive was taken from the medical file. This clinical diagnosis was based on clinical parameters, imaging and laboratory results, and disease course as follows: a patient was considered as COVID-19 positive with either a positive RT-PCR test or a negative RT-PCR test and clinical symptoms and/or history of contact with COVID-19 patients and/or highly suggestive chest CT findings.

### Image analysis

All CT scans were scored using CO-RADS and CTIS. For CO-RADS the level of suspicion of COVID-19 infection was graded from very low (CO-RADS 1) up to very high (CO-RADS 5). The CTIS was based on the extent of lobar involvement of typical COVID-19 CT findings [10,15]. This extent was evaluated by scoring the percentages of involvement in each of the five lobes (CTIS 1 for <5% involvement, score 2 for 5–25% involvement, score 3 for 26–50% involvement, score 4 for 51–75% involvement, and score 5 for >75% involvement), resulting in a total score on 25 [16]. Three CTIS-based categories were defined as: 0–2, 3–6, >6 representing respectively mild, moderate, and severe lung involvement. All CT scans were scored by two readers (reader 1: a senior thoracic radiologist with 21-year experience and reader 2: a resident radiologist with one-year experience). Discrepant cases were evaluated by a third reader (a senior thoracic radiologist with 10-year experience). Before reviewing the CT-scans all readers studied literature of CT imaging features of COVID-19 pneumonia [9,10,12,13,14,15]. Readers were blinded to the lab results, radiology reports, and medical files.

### Statistical analysis

Using the results of the image analysis the following ‘imaging groups’ were defined:

- CO-RADS 4/5 (CO-RADS score of 4 or 5).
- Moderate or Severe CTIS (CTIS score >2/25).
- Severe CTIS (CTIS score >6/25).
- Mixed severe CTIS and/or CO-RADS 4/5.

Diagnostic performance of each ‘imaging group’ for COVID-19 infection was evaluated by calculating sensitivity, specificity, positive predictive value (PPV), negative predictive value (NPV), accuracy, and area under curve (AUC) using each of RT-PCR and the final clinical diagnosis as the gold standard. All statistical analysis procedures were performed using SPSS version 24.0 (IBM, Armonk, NY). Inter-observer agreement was measured using κ-values and interpreted according to Landis and Koch [17].

## Results

Two hundred patients with CT scans were included and analyzed according to CO-RADS and the CTIS; 96 (48%) were male and 104 (52%) were female; mean age was 67.30 ± 17.11 years (***Table 1***). Of note, 94 repeated PCR tests were performed within the 48-hour time interval, finally resulting in 68 positive (34.0%) and 132 (66.0%) negative test results. Inter-observer agreement of the first two readers for CTIS and CO-RADS scores were 0.636 and 0.613 respectively. Distribution of scans with a positive RT-PCR test showed a high number of scans with a high CO-RADS score and a severe CTIS (***Table 1***), whereas cases with a negative RT-PCR test showed high numbers of low CO-RADS and mild CTIS scores.

**Table 1:** Patient characteristics and distribution of CTIS and CO-RADS on chest CT according to nasopharyngeal swab RT-PCR findings.


		PATIENTS WITH RT-PCR AND CHEST-CT (n = 200)	COVID-19 + ON RT-PCR (n = 68)	COVID-19 – ON RT-PCR (n = 132)

Age (years)*		67.30 (±17.11)	65.02 (±18.53)	68.75 (±16.00)

Male gender		96 (48.0)	34 (50.0)	62 (47.0)

CT-involvement score	Mild (0–2)	100 (50.0)	11 (16.2)	89 (67.4)

	Moderate (3–6)	24 (12.0)	5 (7.4)	19 (14.4)

	Severe (>6)	76 (38.0)	52 (76.5)	24 (18.2)

CO-RADS score	1	66 (33.0)	4 (6.8)	62 (47.0)

	2	26 (13.0)	2 (2.9)	24 (18.2)

	3	28 (14.0)	2 (2.9)	26 (19.7)

	4	19 (9.5)	10 (14.7)	9 (6.8)

	5	61 (30.5)	50 (73.5)	11 (8.3)


Data are numbers with percentages in parentheses. * Data are means with ± standard deviation in parentheses.

An absolute agreement of CO-RADS scores and CT-involvement scores was seen in 141/200 (70.5%) and 155/200 (77.5%) of cases, respectively, and 29/200 (14.5%) of the observations had CO-RADS scores that varied between 1 and 2 (low suspicion of COVID-19) or between 4 and 5 (high suspicion of COVID-19). Thus, for 171/200 (85.5%) of the observations, there was an agreement for the diagnosis of COVID-19 using CO-RADS scoring.

### CT diagnosis of COVID-19

The results of the ‘imaging groups’ for diagnosing COVID-19 using RT-PCR test as the gold standard are shown in ***Table 2***. In general, all ‘imaging groups’ had an excellent negative predictive value (NPV) (range 108/124 [87.1%] to 112/120 [93.3%] respectively). The highest area under curve (AUC: 0.865) and accuracy (86.0%, 172/200) was reached in the CO-RADS 4/5 group with a sensitivity of 60/80 (88.2%) and a specificity of 112/132 (84.9%) (***Table 2***). The mixed severe CTIS and/or CO-RADS 4/5 group showed a sensitivity of 60/68 (88.2%) and a specificity of 107/132 (81.1%). We reviewed the medical files of the patients with CT false-positive and false-negative findings versus RT-PCR in the CO-RADS 4/5. There were eight false negative and 20 false positives. In four of the eight false negatives, the first RT-PCR test was negative, and the second RT-PCR test performed within 48 hours was positive. One of these four cases had a second CT (of the abdomen) one week later, showing imaging findings consistent with COVID-19 in the lung bases. The other four cases included patients that presented clinically with none or very light (upper) respiratory tract symptoms (n = 2), or with mainly abdominal complaints (pain, diarrhea) (n = 1), or who underwent chest CT the first day of symptom onset (n = 1).

**Table 2:** Diagnostic value of CTIS and CO-RADS on chest CT for the diagnosis of COVID-19 infection using RT-PCR as the standard of reference.


	TP	FP	TN	FN	SENSITIVITY* (95% CI)	SPECIFICITY* (95% CI)	PPV* (95% CI)	NPV* (95% CI)	ACCURACY* (95% CI)	AUC (95% CI)

Moderate or Severe CTIS	57	43	89	11	83.8 (72.9–91.6)	67.4 (58.7–75.3)	57.0 (50.4–63.38)	89.00 (82.3–93.4)	73.0 (66.3–79.0)	0.756 (0.686–0.826)

Severe CTIS	52	24	108	16	76.5 (64.6–85.9)	81.8 (74.2–88.0)	68.4 (59.6–76.1)	87.1 (81.4–91.3)	80.0 (73.8–85.3)	0.791 (0.722–0.778)

CO-RADS 4/5	60	20	112	8	88.2 (78.1–94.8)	84.9 (77.6–90.5)	75.0 (66.5–81.9)	93.3 (87.9–96.4)	86.0 (80.4–90.5)	0.865 (0.809–0.922)

Mixed Severe CTIS and/or CO-RADS 4/5	60	25	107	8	88.2 (78.1–94.8)	81.1 (73.3–87.3)	70.6 (62.5–77.5)	93.0 (87.4–96.3)	83.5 (77.6–88.4)	0.846 (0.787–0.906)


TP: true positive, FP: false positive, TN: true negative, FN: false negative, PPV: positive predictive value, NPV: negative predictive value, AUC: area under the curve, CI: confidence interval. Unless otherwise specified data are numbers. CTIS: CT involvement score. * Data are percentages.

**Figure 1:**
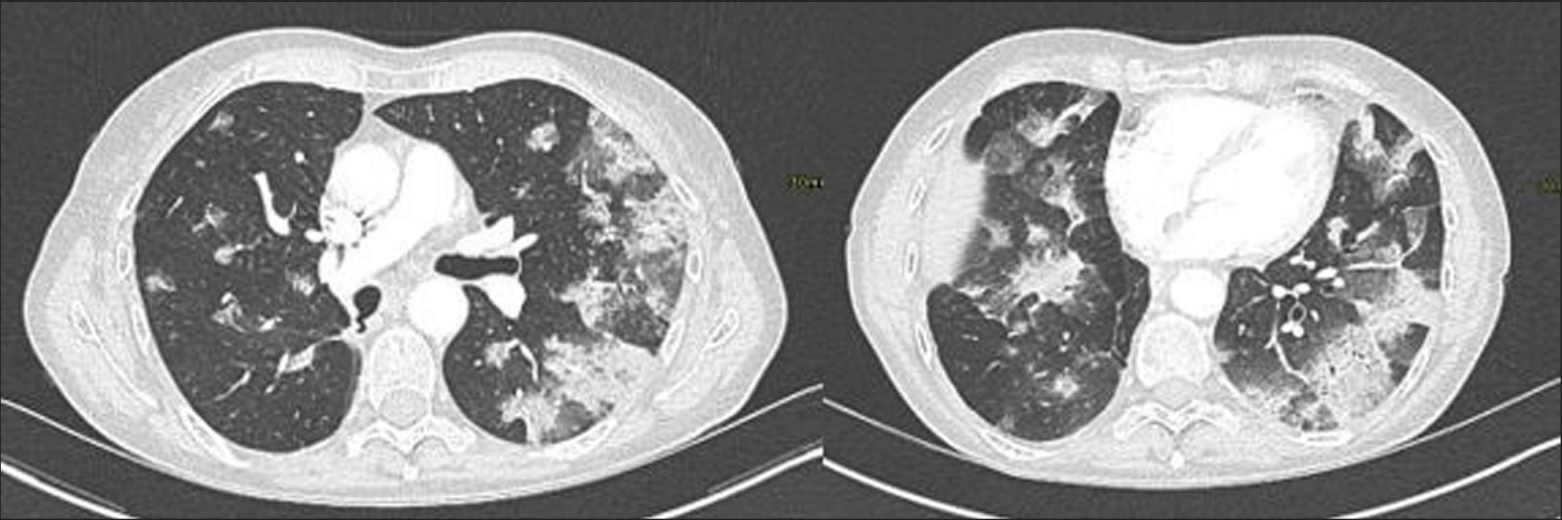
False-positive CO-RADS chest CT for COVID-19 when compared to RT-PCR test but considered true positive by treating clinician: 74-year-old man with four weeks of diarrhoea and two weeks of coughing. CO-RADS score of 5. At the time of scanning, no residual respiratory symptoms or fever were present and RT-PCR test was negative three times.

**Figure 2:**
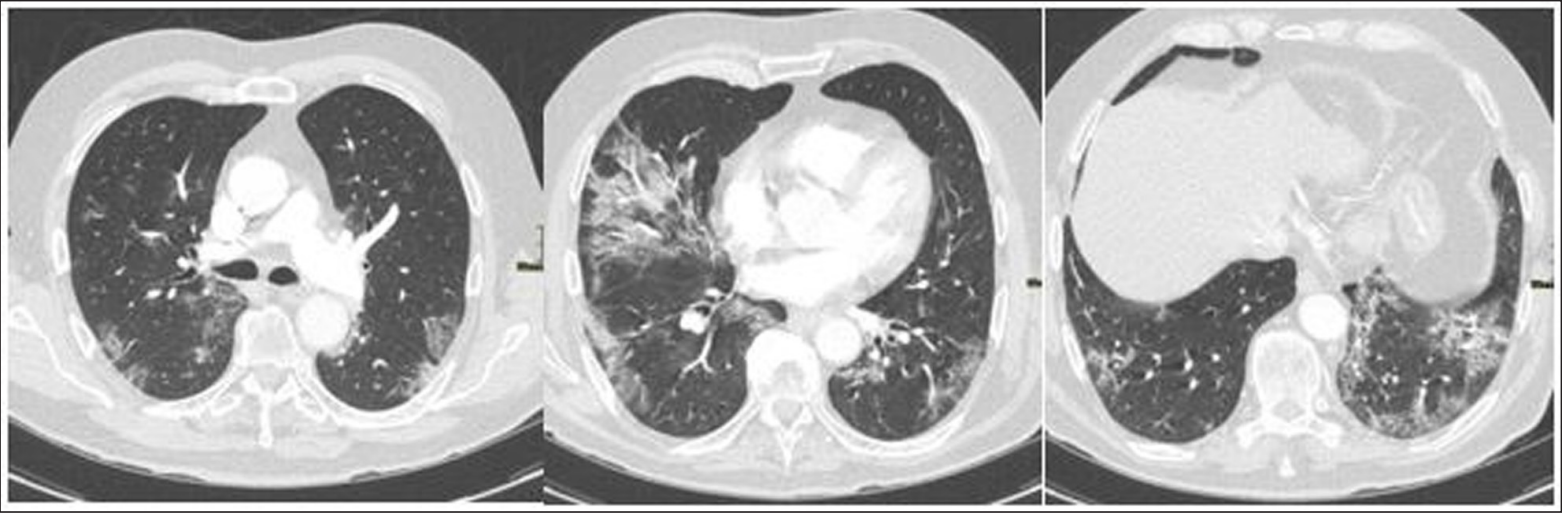
Positive CO-RADS chest CT for COVID-19 with a negative RT-PCR test: This 42-year-old patient was tested negative for COVID-19 with RT-PCR and showed multiple symptoms for COVID-19 (cough, fever, and anosmia). The chest CT showed multiple ground glass opacities and consolidation areas with crazy-paving patterns and was scored as CO-RADS 4. This patient was also considered as positive for COVID-19 by the clinician.

**Figure 3:**
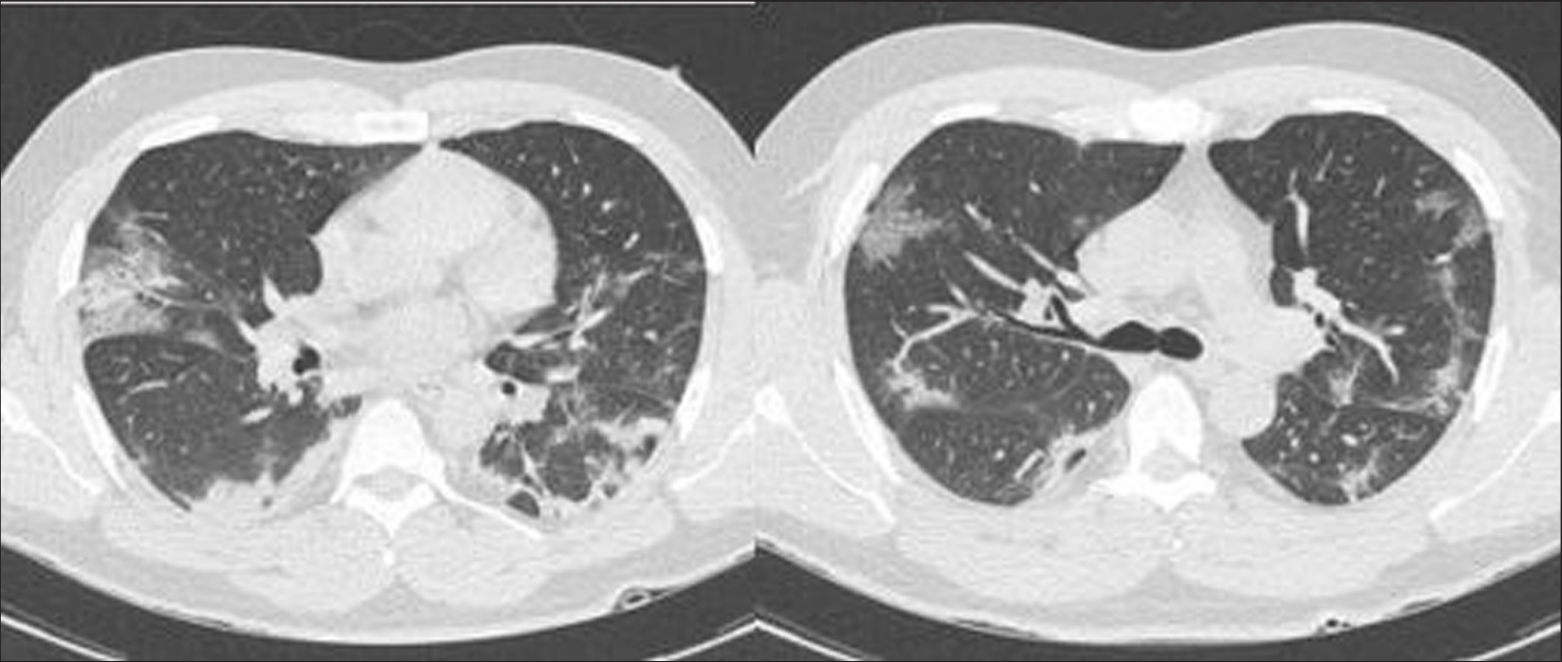
False-positive CO-RADS chest CT for COVID-19 when compared to RT-PCR test: 61-year-old woman with metastatic adenocarcinoma under chemotherapy. CO-RADS score of 4. No significant change on CT when comparing to previous scans (not available to the reader at the time of scoring).

Ten of the 20 false-positives were considered as COVID-19 positive by the treating physician, based on clinical findings and disease course (***Figures 1*** and ***2***). All of these cases were sufficiently ill to be admitted to the hospital and even the intensive care unit (two patients). The remaining 10 false positives were eventually considered negative by the clinician. Two of these 10 cases were known with another pneumonia or metastatic disease with superinfection and would not have been labeled as chest CT CO-RADS 4/5 positive if the patient medical files would have been available to the radiologists scoring the chest CT examinations (***Figure 3***). The remaining eight false positives were cardiac failure (n = 2), COPD exacerbation (n = 1), aspiration pneumonia (n = 1), and bacterial pneumonia (n = 4). As a result, using the clinical diagnosis as the gold standard, the accuracy for the diagnosis of COVID-19 increased in all chest CT ‘imaging groups’ (***Table 3***).

**Table 3:** Diagnostic value of CTIS and CO-RADS on chest CT for the diagnosis of COVID-19 infection using the final clinical diagnosis as the standard of reference.


	TP	FP	TN	FN	SENSITIVITY* (95% CI)	SPECIFICITY* (95% CI)	PPV* (95% CI)	NPV* (95% CI)	ACCURACY* (95% CI)	AUC (95% CI)

Moderate or Severe CTIS	68	32	89	11	86.1 (76.5–92.8)	73.6 (64.8–81.2)	68.0 (60.9–74.3)	89.00 (82.2–93.4)	78.5 (72.2–84.0)	0.798 (0.734–0.862)

Severe CTIS	62	14	107	17	78.5 (67.8–86.9)	88.4 (81.3–93.5)	81.6 (72.75–88.0)	86.3 (80.4–90.6)	84.5 (78.7–89.2)	0.835 (0.772–0.897)

CO-RADS 4/5	70	10	111	9	88.6 (79.5–94.7)	91.7 (85.3–96.0)	87.5 (79.4–92.7)	92.5 (86.9–95.8)	90.5 (85.6–94.2)	0.902 (0.852–0.951)

Mixed Severe CTIS and/or CO-RADS 4/5	70	14	107	9	88.6 (79.5–94.7)	88.4 (81.4–93.5)	83.3 (75.2–89.2)	92.2 (86.5–95.7)	88.5 (83.3–92.6)	0.885 (0.833–0.937)


TP: true positive, FP: false positive, TN: true negative, FN: false negative, PPV: positive predictive value, NPV: negative predictive value, AUC: area under the curve, CI: confidence interval. Unless otherwise specified data are numbers. CTIS: CT involvement score. * Data are percentages.

## Discussion

In this retrospective study we pursued to evaluate the accuracy of CT using the CO-RADS and CTIS for suggesting COVID-19 infection compared to RT-PCR and clinical diagnosis. CO-RADS has shown good results for the detection of COVID-19 in symptomatic patients, but less good results in asymptomatic patients [12,18]. Meanwhile CTIS has been used to evaluate the extent pulmonary involvement of COVID-19 and shown to be correlated with age, inflammatory biomarkers and severity of clinical categories [14], but never tested as a diagnostic tool.

In our study, the ‘imaging group’ CO-RADS 4/5 performed best with AUC of 0.865 when compared to RT-PCR and 0.902 when compared to the final clinical diagnosis. These findings are consistent with the original CO-RADS study reporting AUC of 0.91 when compared to RT-PCR and 0.95 when compared to the final clinical diagnosis (12). The CTIS performed well in our study but showed less good results than CO-RADS; this might be explained by the fact that CTIS is more directed at classifying pulmonary extent of the disease rather than diagnosing the disease itself. Combining both scoring systems showed no clear improvement for diagnosing COVID-19. While CTIS showed relatively good diagnostic value for COVID-19 in our cohort, we advise keeping it for extent assessment and continue using CO-RADS for diagnostic purpose on chest CT.

The PPV of both CO-RADS and CTIS (75.0% and 68.4% respectively) is less high, which should prompt clinicians to consider the clinical findings, laboratory results, and disease course before ascertaining the diagnosis of Covid-19 when the CT findings are suggestive. Actually, false-negative chest CT are mainly explained by imaging within the first day of the clinical disease course or COVID-19 infection with no or minor respiratory symptoms. About half of the false-positive chest CT scans compared to RT-PCR were associated with other pneumonias and cardiac failure.

Several limitations to this study have to be addressed. First, referral bias in this retrospective study was inevitable as patients examined by CT naturally tended to be more sick. Second, observer bias could be possible. Although blinded for RT-PCR results, readers were aware of the level of suspicion for COVID-19 in this cohort. Third, the study was performed in a COVID-19 high-prevalence area. Finally, disease spread throughout Belgium and the rest of Europe distinctly from the seasonal influenza outbreak, thus the number of overlapping patterns due to other viruses was limited.

In summary, this study performed in a COVID-19 high-prevalence area found that CO-RADS chest CT was highly accurate for detecting COVID-19 pneumonia and showed better results than CTIS. Combining CO-RADS and CTIS showed no improvement in accuracy.
